# Flexible Analog Search with Kernel PCA Embedded Molecule Vectors

**DOI:** 10.1016/j.csbj.2017.03.003

**Published:** 2017-03-24

**Authors:** Stefano Rensi, Russ B. Altman

**Affiliations:** Department of Bioengineering, Stanford University, Shriram Center Room 213, 443 Via Ortega MC 4245, Stanford, CA 94305, United States

## Abstract

Studying analog series to find structural transformations that enhance the activity and ADME properties of lead compounds is an important part of drug development. Matched molecular pair (MMP) search is a powerful tool for analog analysis that imitates researchers' ability to select pairs of compounds that differ only by small well-defined transformations. Abstraction is a challenge for existing MMP search algorithms, which can result in the omission of relevant, inexact MMPs, and inclusion of irrelevant, contextually dissimilar MMPs. In this work, we present a new method for MMP search that returns approximate results and enables flexible control over abstraction of contextual information. We illustrate the concepts and mechanics of our method with a series of exemplar MMP queries, and then benchmark search accuracy using MMPs found by fragment indexing. We show that we can search for MMPs in a context dependent manner, and accurately approximate context independent fragment index based MMP search over a range of fingerprint and dataset conditions. Our method can be used to search for pairwise correspondences among analog sets and bolster MMP datasets where data is missing or incomplete.

## 1 Introduction

### 1.1 Analog Search is Important for Lead Optimization

Successful optimization of lead compounds requires the iterative application of structural modifications that yield favorable changes in target activity profiles and ADMET properties. Traditionally, this process has been the sole domain of medicinal chemistry teams. Researchers assembled analog series data by hand, guided by their knowledge of compounds that had been synthesized and tested within their organization. They would then generate hypotheses using techniques such as Free-Wilson analysis [1], Hansch analysis [2], Topliss schemes [3], and Craig plots [4]; and combine data driven insights with expertise to prioritize compounds for synthesis and testing in each design iteration. Today, the overall process of lead optimization is similar, but the volume chemical information in corporate and public databases is too large for development teams to process without computational support. The challenge has driven innovation in search and index of analog sets in chemical libraries.

### 1.2 MMPs and MMP Search Are Useful Computational Constructs

Matched molecular pairs (MMPs) are a concept in analog analysis that formalizes a particular type of analog relationship: two molecules that differ at a single site by a specific transformation. MMP analysis has been successfully used to study the effects of transformations on ADME properties [5], [6], solubility [7], chemical activities [8], as well as bioisosterism and activity cliffs [9], [10], [11]. A diverse set supervised and unsupervised approaches to MMP search have been described using 2D fingerprints [12], maximum common substructure alignment [9], [12], [13], SMILES/SMARTS editing [5], [7], [14], [15], and molecule fragmentation [16], [17]; though many are difficult to evaluate because the software or detailed descriptions of the underlying algorithms are not in the public domain. The unsupervised algorithm published by Hussain and Rea is currently the most widely used [17]. It efficiently discovers all MMPs in a dataset by exhaustive fragmentation at non-ring single bond sites followed by indexing of cores and substituents into a bipartite data structure.

### 1.3 MMP Search is Limited by Abstraction

The key limitation of MMP search is abstraction. By definition MMPs group together pairs of molecules with the exact same transformation. Unfortunately, this excludes many near-MMPs relevant to the analysis. Griffen et al. gave the example of using a methyl sulfone addition for an analysis where the effect of the desired ethyl sulfone addition had not been previously observed [18]. Furthermore, transformation indexing is done in a context independent manner; all scaffolds undergoing a particular transformation are grouped together. We refer to the molecular context as the common substructure and transformation site shared by two analogs. For example, for the methylation of L-Dopa into 3-O-methyl-L-Dopa, the transformation would be the replacement of hydrogen by a methyl group, and the context would be the 3-hydroxyl substituent of L-Dopa. Papadatos et all demonstrated how much context matters by comparing context specific and global distributions of transformation effects on hERG inhibition [19]. We also note that the issues arising from the exactness of fragment indexing and the looseness of scaffold grouping exacerbate each other. As we narrow the rage of contexts to be more specific, we would also expect to find fewer MMPs that match any particular transformation. Thus there is a need for methods of querying MMPs that can return approximate matches and flexibly integrate varying amounts of contextual information.

### 1.4 We Propose More Flexible MMP Search

In this work, we develop a method for flexible MMP search that allows varying levels of abstraction. Our method represents chemical transformations as vector differences, in a manner similar to Sheridan et al. [12], with the difference that we use dimensionality reduction techniques to embed molecule vectors in continuous space and reformulate MMP search as a supervised query. Our queries return approximate MMPs, and our continuous vectors allow us dynamic control over the level of abstraction at which we search. First we develop our search method and demonstrate its mechanics on an exemplar analog series of nucleic acids and nucleosides. Second, we benchmark MMP recall on datasets of varying size and diversity using embedded molecule vectors derived from several different underlying fingerprint representations. Finally, we summarize our results and highlight some considerations for using our method.

## 2 Methods

### 2.1 2D Fingerprint and MMP Generation

We generated 2D chemical fingerprints and MMPs using RDKit for Python 3.0 [20]. We encoded chemical structures using four types of fingerprints. Extended connectivity fingerprints (ECFP_6), atom pair fingerprints (APFP), topological torsion fingerprints (TTFP), and RDKit Path Fingerprints (RDFP). For each fingerprint type, we used the default settings as inputs, and returned fingerprints as unfolded, sparse vectors. For MMPs we used a max fragment length of 10 atoms, and max fragment ratio of 0.3. We implemented vector generation in R-3.03.

### 2.2 Molecule Vector Generation

We embedded 2D fingerprints in continuous space using kernel principal components analysis (KPCA), a non-linear dimensionality reduction technique similar to principal components analysis (PCA) and multidimensional scaling [21]. We used Tanimoto similarity (aka Jaccard index) as our kernel function. Vector generation can be broken down into three steps:

(1) **Compute the Tanimoto matrix**: Compute a kernel matrix of Tanimoto similarities *T*(*X*, *X*) over all pairs of data instances (*x*_*i*_, *x*_*j*_) ∈ *X* , *X*.
  $$T\left(x_{i},x_{j}\right)=\frac{x_{i}\cap x_{j}}{x_{i}\cup x_{j}}$$
(2) **Solve the Eigenvalue problem**: Factor the Tanimoto matrix by solving the eigenvalue problem. Here *ϕ*(*X*) denotes the data represented as continuous vectors, and the matrices *Q* and *Λ* are the eigenvectors and eigenvalues of the Tanimoto similarity matrix.
  $$\phi \left(X\right)\cdot \phi {\left(X\right)}^{T}=\mathrm{Q\Lambda}Q^{T}=T\left(X,X\right)$$
  $$\phi \left(X\right)=Q\Lambda^{1/2\;}$$
(3) **Embed the data in continuous space**: Compute a vector of Tanimoto distances and multiply by the eigenvectors and inverse square root of the eigenvalues to project the data instances into the principal component space.
  $$\phi \left(x_{i}\right)=T\left(x_{i},X\right)\cdot Q\Lambda^{-1/2}$$

Like classical PCA, the features returned by KPCA are orthogonal and ordered by their explanatory power. Similar considerations for dimensionality reduction also apply. Unlike classical PCA, KPCA is non-linear and dot products of Tanimoto KPCA embedded vectors approximate Tanimoto similarities.

### 2.3 Analog Search

We have broken down our method into four component concepts that build on each other into approximate MMP search: the analog score, basic search, basic feature selection, and uncoupled feature sets. Our method was implemented using built in functions R-3.0.3.

### 2.4 Analog Score

The analog score is a measure of similarity between *relationships*. We score the similarity of pairwise relationships {*a*, *b*} and {*c*, *d*} with the following formula:

$$\mathrm{Score}\left(a:b∷c:d\right)=\frac{\left(b-a+c\right)∙d}{\left\|\left(b-a+c\right)\right\|\left\|d\right\|}$$

We point out that the algebraic structure of the score gives a non-intuitive logical equipoise *a* : *b* : : *c* : *d* ≡ *a* : *c* : : *b* : *d*.

### 2.5 Basic Search

Our search objective is to find the molecules that correctly or approximately complete an MMP as an analogy:

$$\mathrm{Given molecules}\;\left\{a,b,c\right\},\mathrm{find}\;d\in D\;\mathrm{such that}\;a:b::c:d$$

To search a list of candidates (*D*), we compute analog scores for the input triple (*a*, *b*, *c*) with each molecule (*d*) in the list, order *D* by score, and return the top *n* results.

### 2.6 Basic Feature Selection

The subset of components used to represent the data is an independent parameter in the search. We call this the dimensionality parameter *p*. While dimensionality reduction is typically done by using the first *p* < *n* principal components, it is not the case that the components must be included or excluded in consecutive order (order of decreasing variance). Thus we introduce the more general notation *ω* ⊆ {1, … , *n*} to denote an active subset of components used to represent the data, and the indicator function *I*(*ω*), where *I*(*ω*)_*jj*_ = 1 if *j* ∈ *ω* else it is 0. For example, if we wish to represent the data using the first three principal components, then *ω* = {*j* : *j* ≤ 3} and *I*(*ω*)_11_ = *I*(*ω*)_22_ = *I*(*ω*)_33_ = 1. The reduced dimension representation of a molecule vector *ϕ*(*x*)_*ω*_ is given by:

$${\phi \left(x_{i}\right)}_{\omega }=\phi \left(x_{i}\right)\cdot I\left(\omega \right)$$

### 2.7 Uncoupled Feature Sets

To flexibly control the level of abstraction at which we search, we decompose query vectors into two components: (a) the difference of two molecules – the relation, and (b) the remaining summand molecule – the target.

$$\underset{\mathrm{query}}{\underbrace{\left(b-a+c\right)}}=\underset{\mathrm{re}\mathrm{lation}}{\underbrace{b-a}}+\underset{\mathrm{target}}{\underbrace{c}}$$

Importantly, we select features independently for relation and target. Specifically, we assign each term a feature selector parameter – *ω*_*r*_ and *ω*_*t*_. For example, we may wish to compute a query with a coarse grained relationship, using only the first three principal components *ω*_*r*_ = {*j* : *j* ≤ 3}, and a more detailed representation of the target, using the first 20 components *ω*_*t*_ = {*j* : *j* ≤ 20}. We compute the query vector using the following formula:

$$\left(b-a\right)\cdot I\left(\omega_{r}\right)+c\cdot I\left(\omega_{t}\right)$$

### 2.8 Search Examples

We provide a series of four examples, centered on the analog series of nucleic acids and nucleosides, to illustrate the analog score, basic search, basic feature selection, and uncoupled feature sets. Our example dataset consists of 1398 small molecule, FDA approved drugs from Drugbank [22], and 635 biologically important metabolites from the KEGGBRITE ontology [23]. We excluded non-human native classes from the KEGGBRITE set such as marine, fungal, and phytochemical compounds. After duplicate and salt removal, we filtered by molecular weight, excluding molecules outside of the range 100–700 to focus on molecules in the “druglike” size range.

For the analog score, we computed scores for all 8 unique analogous nucleobase pair permutations e.g. {*a* : *g* ∷ *c* : *t*, *a* : *t* ∷ *g* : *c*, …}. For basic search, we queried a set of analogous nucleobase/nucleoside MMPs, and return the top 5 search results as well as the search rank of the correct nucleoside. For basic feature selection, we computed the same set of queries, and tracked the search ranks of all nucleosides as we varied the number of principal components used to represent the data from 3 to 150. For uncoupled feature selection, we independently varied the feature selector parameters *ω*_*r*_ and *ω*_*t*_ each over the range 4–50 and recorded the search rank of the correct nucleoside. We computed analog scores and basic search using the first 20 principal components.

### 2.9 Approximate Context Independent MMP Search Benchmarking

We tested the accuracy of approximate MMP recall with the following protocol. For each dataset: (a) find a set of true positive analogous MMPs using fragment indexing, compute completion queries for all unique analogous pairs, and record the search rank of the correct top result in each case. We executed this over a set of 72,251 compounds, grouped into 102 activity class datasets, and further consolidated into Easy, Intermediate, and Difficult superclasses on the basis of dataset size and diversity [24], [25]. The number of compounds and mean ECFP_4 Tanimoto coefficient for each superclass reported by Jasial et al. is shown in Table 1 [25]. We downloaded structures from ChEMBL in SMILES format, and removed salts.

We used the following feature selection strategy to approximate the context independence of fragment indexing MMP search. Given molecule vectors *a* , *b*, we set *ω*_*r*_ = {*j* : *a*_*j*_*b*_*j*_ < 0}. In other words, we represent the transformations using features for which the molecules that define the transformation differ in sign. For target molecules we used the full rank representation *ω*_*t*_ = {*j* : *λ*_*j*_ > 10^− 10^}.

## 3 Results

### 3.1 Molecule Vector Generation

**Fig. 1:**
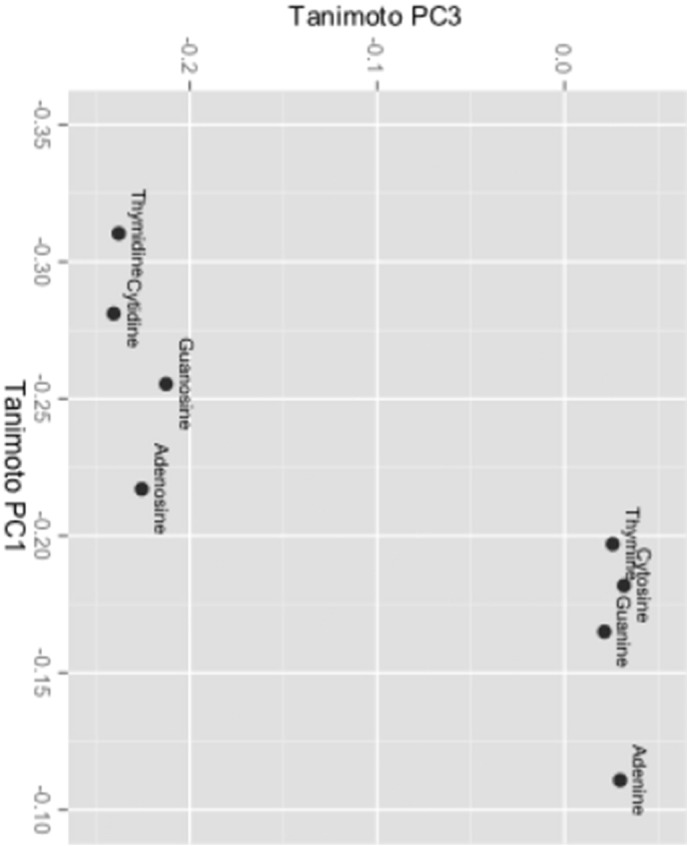
**Nucleic bases and nucleosides in continuous space**. Plot showing the arrangement of nucleic bases and nucleosides in continuous space generated by KPCA. Coordinates along the first and third principal component axes are shown. Analogous nucleobase/nucleoside pair correspondences map to corresponding spatial orientations.

Fig. 1 shows a plot of the nucleobases and nucleosides along the first and third principal axes. Analogous nucleobase/nucleoside MMPs have similar spatial orientations.

### 3.2 Analog Score

**Fig. 2:**
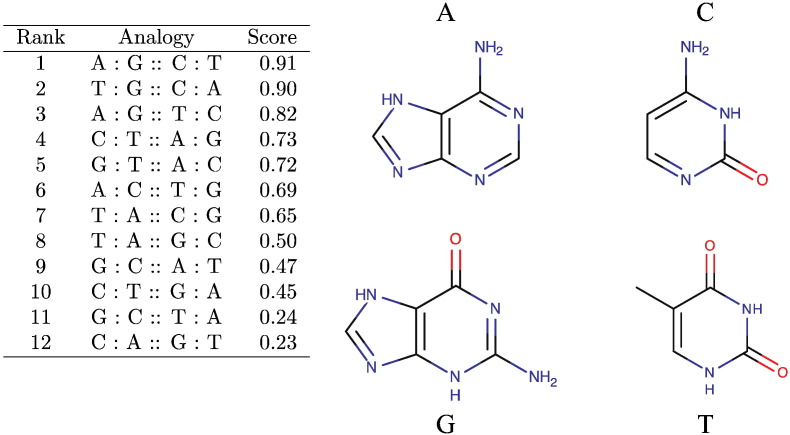
**Analog score**. The structures of nucleic acids and scores for all unique base pair analogies. The analog score has the non-intuitive equipoise *score*(*a* : *b* : : *c* : *d*) = *score*(*a* : *c* : : *b* : *d*).

The sorted scores of the 8 unique possible analogous nucleobase pair arrangements are shown in Fig. 2 along with the structures of the nucleobases: adenine, guanine, cytosine, and thymine. The top scoring pairwise alignment gives the correct purine/pyrimidine, hydrogen bond donor/acceptor correspondences.

### 3.3 Basic Search

**Fig. 3:**
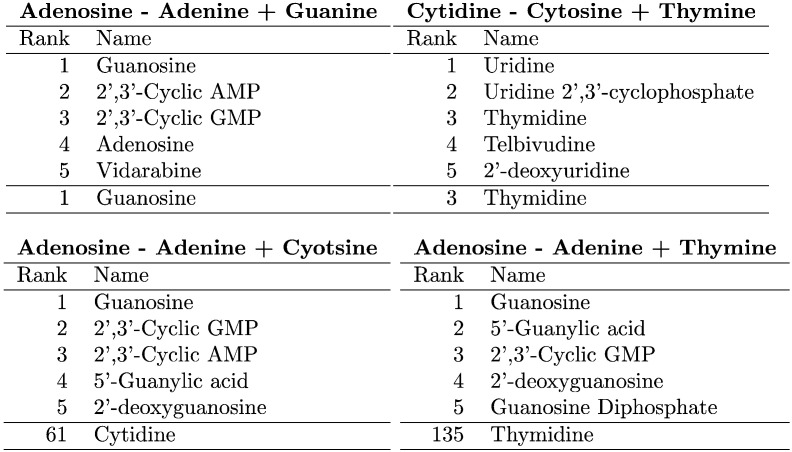
**Basic search**. Search results for four nucleobase/nucleoside MMP queries, each with a different degree of contextual similarity between analogous pairs. **(a)** Query for Guanosine across H-donor/acceptor context. **(b)** Query for Thymidine across H-donor/acceptor contexts. **(c)** Query for Cytidine across purine/pyrimidine contexts. **(d)** Query for Thymidine across both H-donor/acceptor and purine/pyrimidine contexts. The rank of each intended top result is shown at the bottom of its table.

Fig. 3 shows the results of 4 basic supervised MMP searches. MMPs queries across hydrogen bond donor/acceptor contexts (*A* : *A*^∗^ ∷ *G* : *G*^∗^) perform better than queries across purine/pyrimidine contexts (*A* : *A*^∗^ ∷ *C* : *C*^∗^), which perform better than queries across both contexts (*A* : *A*^∗^ ∷ *T* : *T*^∗^).

### 3.4 Basic Feature Selection

**Fig. 4:**
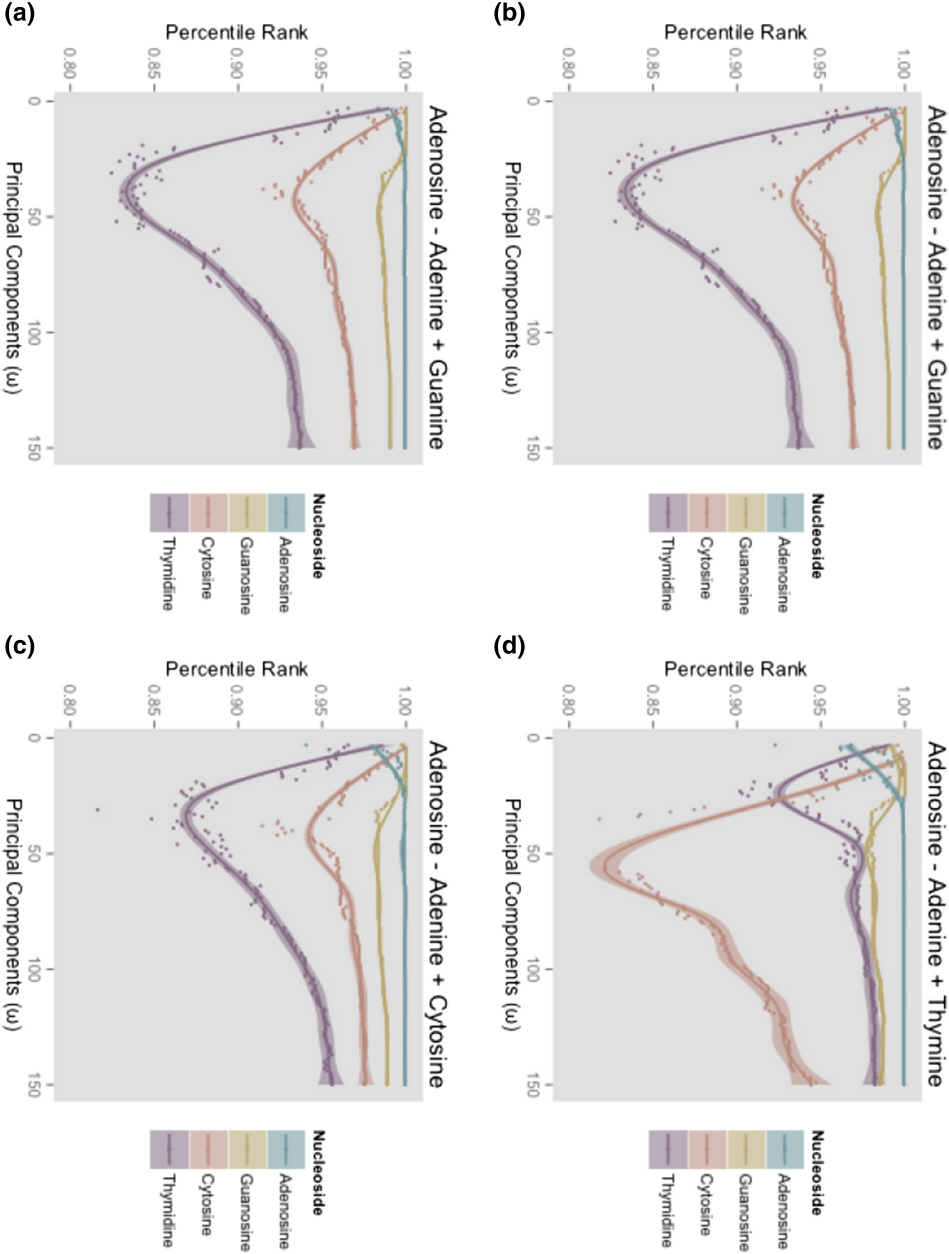
**Basic feature selection.** The four nucleobase/nucleoside MMP queries computed using a range of principal components to represent the data. **(a)** Query for Guanosine across H-donor/acceptor context. **(b)** Query for Thymidine across H-donor/acceptor contexts. **(c)** Query for Cytidine across purine/pyrimidine contexts. **(d)** Query for Thymidine across both H-donor/acceptor and purine/pyrimidine contexts. The x-axis on each plot indicates the number of principal components used to represent the data, and the y-axis shows search rank percentile. For each query, the search ranks of all nucleosides are shown.

Fig. 4 shows the effect of dimensionality reduction on search. Plots display the search ranks of all four nucleosides as we vary the number of principal components in each search example. All nucleosides are highly ranked at low dimension. Rankings diverge as dimension increases. At high dimension the nucleoside used to construct the query saturates at the top result. Basic feature selection was not able to recover queries across hydrogen bond donor/acceptor contexts (Fig. 4(c), (d)). We did not observe any meaningful change in rankings above 150 principal components.

### 3.5 Uncoupled Feature Sets

**Fig. 5:**
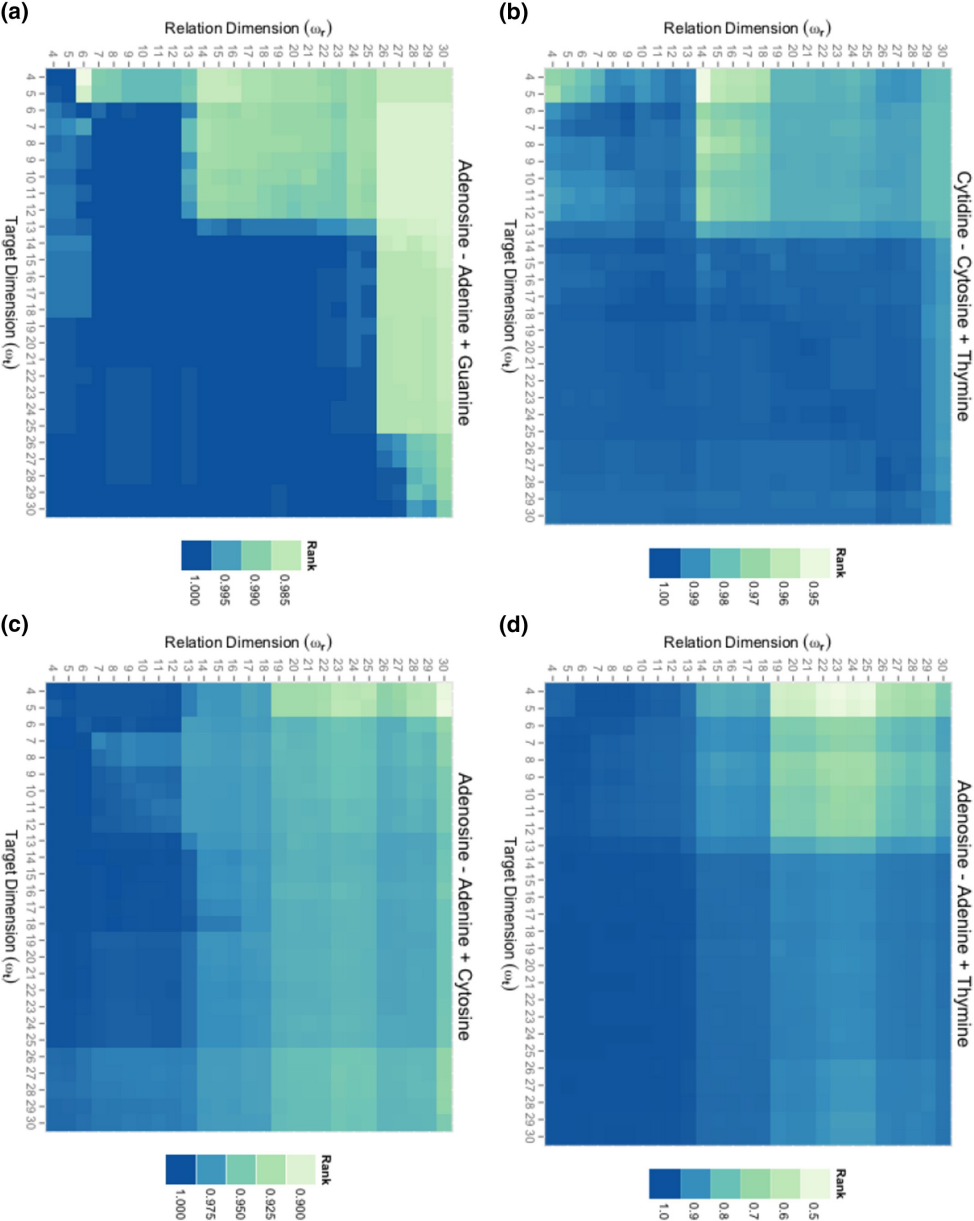
**Uncoupled feature selection.** The four nucleobase/nucleoside MMP queries computed using varying numbers of principal components to represent transformations/relation (*ω*_*r*_) and molecules/target (*ω*_*t*_). **(a)** Query for Guanosine across H-donor/acceptor context. **(b)** Query for Thymidine across H-donor/acceptor contexts. **(c)** Query for Cytidine across purine/pyrimidine contexts. **(d)** Query for Thymidine across both H-donor/acceptor and purine/pyrimidine contexts. For each query, the x-axis and y-axis respectively indicate relation (*ω*_*r*_) and target (*ω*_*t*_) vector dimensions used to compute the query. The color of each cell indicates the rank of the intended result for that query.

Fig. 5 shows the search rank of the correct nucleoside for each query as we independently vary the number of principal components used to represent relationships (transformations) and targets (molecules). Computing the relationship/transformation vector at low dimension (4–12), and adding it to a high dimension target/molecule vector (20–25) rescued queries across purine/pyrimidine contexts (Fig. 5(c), (d)), and did not significantly diminish performance on queries across hydrogen bond donor/acceptor contexts (Fig. 5(a)(b)).

### 3.6 Approximate Context Independent MMP Search Benchmarking

**Fig. 6:**
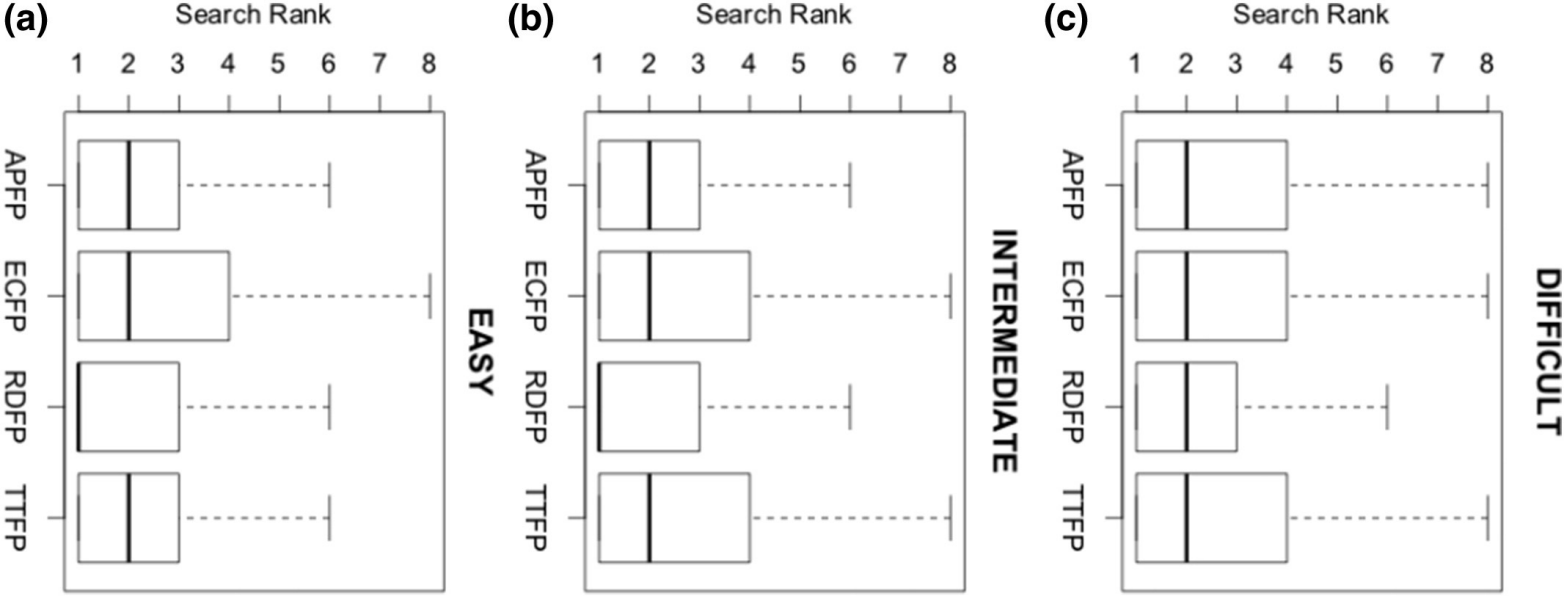
**Context independent search benchmarking.** MMP recall over 102 ChEMBL activity classes for continuous vectors derived from different underlying fingerprint types. Activity classes are grouped into **(a)** “Easy”, **(b)** “Intermediate”, and **(c)** “Difficult” super classes on the basis of size and diversity. For each condition, the box and whisker plot shows the distribution of the search rankings for the correct top result.

Fig. 6 shows the distribution of search ranks assigned to the correct top result in MMP queries for continuous vectors derived from four 2D fingerprint types: APFPs, ECFPs, RDFPs, and TTFPs. Note that we report the absolute rather than the percentile rank. We computed 44,371 (mean = 2016/class), 1,011,564 (mean = 20,231/class), and 4,958,361 (mean = 165,288/class) unique MMP queries for activity class datasets in the Easy, Intermediate, and Difficult superclasses. The distribution of absolute ranks did not change significantly relative to the diversity and size of the datasets in each superclass.

## 4 Discussion

### 4.1 Analog Score Is a Way to Test Similarity of Relationships

The analog score measures the similarity of chemical transformations. Our use of vector differences is similar in concept to the T-Analyze program [12] with the difference that we have algebraically rearranged the scoring function.

$$\underset{T-\mathrm{Analyze}}{\underbrace{b-a\approx d-c}}⟺\underset{\mathrm{Analog Score}}{\underbrace{b-a+c\approx d}}$$

Our function is conceptually similar to supervised MMP queries where a specified transformation is applied to a template molecule [7], [26]. However, we can run in unsupervised mode where we exhaustively compute all pairwise analogies among a set of molecules; or supervised mode, in which one or more of the molecules are specified in advance and held constant. The results of unsupervised mode show some of the flexibility of vector based MMP search. The analogous transformation *A* : *C* ∷ *G* : *T* would not be found by fragmentation at acyclic bonds, but here it is an equivalent top result.

### 4.2 Supervised MMP Search Returns Ordered Lists of Similarly Related Molecules; Context Dependence is a Challenge

MMP search returns lists of near MMPs. In Fig. 3(a), (b), we return closely related, approximate MMP molecules along with the correct nucleoside. We show unsuccessful searches in Fig. 3(c), (d) to demonstrate that transformation vectors encode information about the molecular contexts in which they occur. Sheridan reported a similar effect where transformation vectors clustered together on the basis of context [12]; here it has a confounding effect on searches across purine/pyrimidine contexts. To recover those examples, we would like to operate closer to the context independent manner of fragment indexing schemes.

### 4.3 Basic Feature Selection Allows Us to Include and Exclude Contextual Information

Fig. 4 shows how feature selection can be used to exclude contextual information, but not without challenges. Adding features in order of variance can be interpreted as moving from a coarse to a fine-grained representation of the data. At low dimension we have excluded a significant amount of contextual information, but retained information about the transformation. The correct nucleosides are highly ranked, but indistinguishable from other nucleosides. This is problematic because we would like to discriminate between nucleosides and other closely related molecules to order search rankings. Conversely, we have enough information at high dimension to resolve nucleosides, but the transformations are now context specific. The problem is that we have two types of entities, transformations and molecules, and we would like to represent them at different levels of abstraction. But this is not permitted by typical modes of feature selection. Fortunately, we can separate the level of abstraction for transformations (relationships) and molecules (targets).

### 4.4 Uncoupling Relation and Target Feature Sets is the Secret Sauce

Decomposing query vectors into relationship and target terms with separate feature sets gives us the flexibility to represent transformations and molecules at different levels of abstraction. Our interpretation of Fig. 4 suggests that the combination of a low dimensional relationship vector, with a high dimensional target vector would recover queries across purine/pyrimidine contexts. This is verified in Fig. 5.

### 4.5 Adaptive Feature Selection Approximates Fragment Index MMP search

With our adaptive feature selection heuristic, we are able to accurately approximate fragment index based MMP search (Fig. 6). Recall of fragment indexed MMPs was robust over a range of dataset size and diversity conditions.

### 4.6 Feature Selection Is the Primary Consideration of Our Work

The key idea in our work is that uncoupled feature sets enable us to represent relationships and targets at different levels of abstraction, and thus achieve a more flexible search capability. However, this raises the question of how one should select feature sets. Our results show our method is quite sensitive to feature selection (Fig. 4, Fig. 5). To answer is difficult because the issue is closely linked to one of how much contextual information should be used in search computations. This is often unclear because what constitutes an equivalent transformation is highly subjective and application specific. Thus, the feature selection strategy should be dictated by the particular needs of the application.

For the demonstration examples, our objective was to illustrate different aspects of our method. We chose a grid search over consecutive feature sets because of its intuitive interpretation of moving from a coarse to a detailed representation of the data. In our examples, we showed that we were able access parameter settings that recovered queries of transformations in dissimilar contexts; but the non-smoothness of search rankings in Fig. 4, Fig. 5 suggests that contextual information is not stored in consecutive feature sets, and the strategy would not generally suffice for context independent search.

For benchmarking, our objective was to approximate context independence. We used a rough heuristic to identify context from information contained in the query vectors. For query molecules *a* and *b*, features that match in sign positively contribute to their similarity score and encode their similar parts (context); and those that do not match negatively contribute and encode their dissimilar parts (transformation).

$$\mathrm{sim}\left(a,b\right)\propto a\cdot b=\underset{\mathrm{context}}{\underbrace{\sum_{\mathrm{match}}\left|a_{j}b_{j}\right|}}-\underset{\mathrm{transformation}}{\underbrace{\sum_{\mathrm{mismatch}}\left|a_{j}b_{j}\right|}}$$

In between full context dependence and independence, there are a range of strategies, not limited to reordering transformation features relative to their expected variance and incorporating information about contextual difference of the target molecule, which can be used to tune searches.

### 4.7 Embedded Vectors Are Only as Good as Underlying Representations

A key consideration is the underlying representation used to encode the molecules. Fig. 6 shows it has a measurable effect on accuracy and robustness of search. We found that properties of the 2D fingerprints, such as the repetition insensitivity of binary ECFPs, were passed on to their continuous vectors. We suspect that the RDKit fingerprint representation performs better because of its property that substructure relationships between molecules correspond to subset relationships between their fingerprints. Fingerprint hyperparameters should also be taken into account. We encoded 2D fingerprints as unfolded sparse vectors because in some cases folding resulted in Tanimoto matrices with negative eigenvalues; which is problematic because kernel functions are required to be positive semi definite, and violation of this constraint can cause computations to break down in unpredictable ways.

### 4.8 Embedding Technique is Another Hyperparameter

Another key consideration is the technique used to embed the data in continuous space. We prefer Tanimoto KPCA because it returns uncorrelated features whose dot products approximate Tanimoto coefficients; but it is just one of a variety of techniques for embedding molecules in continuous space. In addition to using alternative similarity metrics and distance-based embedding methods, neural network embedded graph convolution fingerprints [27], [28] are a new type of continuous representation that has shown superior QSAR performance, and could be used with our method. Our method is adaptable to any continuous coordinate representation where the following condition is met: similar chemical structure transformations yield similar transformation vectors. This corollary of linear algebraic consistency should be kept in mind because it is not necessarily the case that differences computed using any set of continuous descriptors should correspond to meaningful chemical structure relationships.

## 5 Conclusion

MMPs are a useful tool to study analog relationships and local QSAR, but current MMP search methods are brittle compared to intuitive notions of what constitutes a matched analog pair. Efficient index based search methods enforce precisely defined context independent transformations that can miss near MMPs relevant to an analysis. Likewise, previous iterations of vector based MMP search enforce strict context dependence and feature set coupling that can fail to group together transformations occurring in different contexts.

We demonstrate a new vector based method for computing approximate MMP queries that allows us to flexibly include or exclude varying degrees of contextual information. We have benchmarked its accuracy for approximate context-independent MMP recall. Given the incompleteness of high confidence assay data in chemical databases, our method can be used to find suitable approximate replacements in cases where the properties of a specific analog found by fragment indexing are uncertain or have not been observed. Our method can also be used to bolster the size of MMP datasets to improve statistical power. Perhaps the most interesting aspect of our work is that kernel embedding can be applied to any symbolic representation that supports similarity computation, opening the prospect of searching and characterizing relationships between non-structural aspects of chemicals such as binding affinity profiles; or even higher order chemical entities such as analog series.

## Figures and Tables

**Table 1:** Size and diversity of MMP search benchmarking dataset superclasses.

	N (mean)	Mean T_c_
Easy	2967 (135)	0.28
Intermediate	25,175 (504)	0.19
Difficult	47,109 (1570)	0.11
